# Oxyfuel Combustion Makes Carbon Capture More Efficient

**DOI:** 10.1021/acsomega.3c05034

**Published:** 2024-01-10

**Authors:** Saeed Talei, Daniel Fozer, Petar Sabev Varbanov, Agnes Szanyi, Peter Mizsey

**Affiliations:** † Institute of Chemistry, University of Miskolc, H-3515 Miskolc, Hungary; ‡ Department of Environmental and Resource Engineering, Technical University of Denmark, 2800 Kgs. Lyngby, Denmark; § Sustainable Process Integration Laboratory − SPIL, NETME Centre, FME, Brno University of Technology − VUT Brno, Technická 2896/2, 616 69 Brno, Czech Republic; ∥ Department of Chemical and Environmental Process Engineering, Budapest University of Technology and Economics, H-1521 Budapest, Hungary; ⊥ Higher Education and Industrial Cooperation Centre, University of Miskolc, H-3515 Miskolc, Hungary

## Abstract

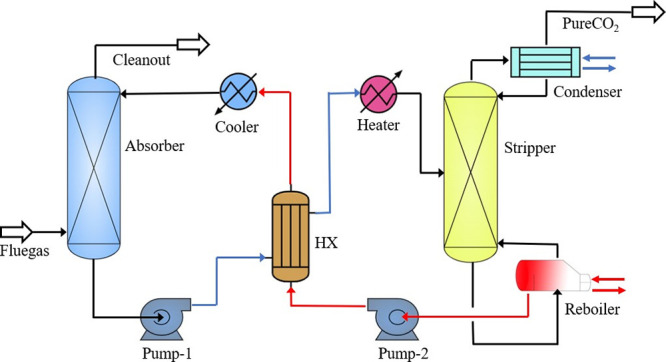

Fossil energy carriers cannot be totally replaced, especially if nuclear power stations are stopped and renewable energy is not available. To fulfill emission regulations, however, points such as emission sources should be addressed. Besides desulfurization, carbon capture and utilization have become increasingly important engineering activities. Oxyfuel technologies offer new options to reduce greenhouse gas emissions; however, the use of clean oxygen instead of air can be dangerous in the case of certain existing technologies. To replace the inert effect of nitrogen, carbon dioxide is mixed with oxygen gas in the case of such air combustion processes. In this work, the features of carbon capture in five different flue gases of air combustion and such oxyfuel combustion where additional carbon dioxide is mixed with clean oxygen are studied and compared. The five different flue gases originate from the gas-fired power plant, coal-fired power plant, coal-fired combined heat and power plant, the aluminum production industry, and the cement manufacturing industry. Monoethanolamine, which is an industrially preferred solvent for carbon dioxide capture from gas streams at low pressures, is selected as an absorbent, and the same amount of carbon dioxide is captured; that is, always that amount of carbon dioxide is captured, which is the result of the fossil combustion process. ASPEN Plus is used for mathematical modeling. The results show that the oxyfuel combustion cases need significantly less energy, especially at high carbon dioxide removal rates, e.g., higher than 90%, than that of the air combustion cases. The savings can even be as high as 84%. Moreover, 100% carbon capture was also be completed. This finding can be due to the fact that in the oxyfuel combustion cases, the carbon dioxide concentration is much higher than that of the air combustion cases because of the inert carbon dioxide and that higher carbon dioxide concentration results in a higher driving force for the mass transfer. The oxyfuel combustion processes also show another advantage over the air combustion processes since no nitrogen oxides are produced in the combustion process.

## Introduction

The use of fossil fuels is still a significant part of the energy mix, and this part is associated with high greenhouse gas emissions.^1,2^ The increasing atmospheric carbon dioxide concentration as a main greenhouse gas is providing major challenges to global sustainability and environmental development, contributing to approximately three-quarters of total emissions from all industries.^3−5^ The CO_2_ concentration in the atmosphere is expected to increase to 550 ppm by 2050 if no action is taken.^6,7^ According to the scenario that was reported by the International Energy Agency, 1 Gt CO_2_/year should be captured by 2030, ramping up to 5 Gt CO_2_/year in 2045.^8^ Therefore, emissions from power plants and high carbon industries should be addressed in the near future.^9^ Energy intensification is a more efficient tool for emission reduction in different industries; however, there are inevitable carbon emissions that should be treated by other approaches such as Carbon Capture Utilization and/or Storage (CCUS). According to international reports, CCUS could play an important role in reducing CO_2_ emissions.^10,11^ Implementation of this process may vary according to several technological parameters, such as the flue gas flow rate, thermodynamic properties, CO_2_ composition, and the origin of flue gas.^12,13^

There are three main options available for the mitigation of greenhouse gas emissions: oxyfuel combustion^14,15^ where the fossil fuel is combusted with oxygen instead of air; air combustion^16,17^ where the combustion process happens in the presence of air; and precombustion^18,19^ where carbon removal occurs prior to combustion.^20^

Carbon capture, as a kind of end-of-pipe treatment method, is a well-studied alternative where amine-based capture is the dominant technology because of its commercial advantage.^21^ Monoethanolamine (MEA) is a useful solvent in industrial applications due to its low cost, high efficiency, and fast absorption rate. It has a relatively high cyclic capacity, meaning 1 mass unit of MEA is capable of binding 0.45 mass of CO_2_.^22^ However, the major inherent drawback of an amine-based CO_2_ capture system is the high energy consumption for solvent regeneration, which can result in a significant decrease of 9–13% in the net plant efficiency.^23,24^ This energy rapidly increases, notably at higher than 90% removal efficiency.^25^ Oxyfuel combustion is the process of burning fuel in a mixture of pure oxygen and recycled flue gas as the fuel combustion reactant instead of using air.^26^ In the case of oxyfuel combustion, there can be two options:

- Clean oxygen is used; in this case, the flue gas contains only CO_2_ and water, and no selective carbon capture is required;^26^
- Oxygen with artificially mixed CO_2_ as inert is used; this alternative is for existing air-based technologies where the inert effect of nitrogen is replaced with the application of CO_2_.^24^

Due to the replacement of nitrogen with recycled CO_2_, there will be a high CO_2_ concentration in the flue gas stream with 90% of CO_2_ on a dry base, and the recovery and sequestration of CO_2_ become much easier.^27,28^ Moreover, the substitution of carbon dioxide instead of nitrogen in this technology helps to prevent the emission of NO_*x*_ as an undesirable product of air combustion technology.^29^

Yushiie et al.^30^ conducted experimental and numerical analysis on the effect of NO_*x*_ formation in the oxyfuel combustion technology, and their results confirmed that NO_*x*_ emissions under CO_2_–O_2_ conditions were lower than those under “air” conditions. Oxyfuel combustion has been approved in several studies, by using different types of fuels, it can reduce NOx emissions.^31−35^ The high concentration of CO_2_ in oxyfuel combustion is beneficial for separating CO_2_ from the flue gas by adsorption or absorption technologies.^36,37^ In this technology, the combination of recycled flue gas and oxygen is utilized for fuel combustion.^38^ This stream is used to reduce the flame temperature and compensate for the volume of missing N_2_ to ensure that there is an acceptable amount of gas to carry the heat through the combustion process.^39^ Life cycle assessment (LCA) is defined as a technique to quantify the potential environmental impacts of product, materials, process, or other measurable activities over its entire life cycle.^40−42^ The LCA of the oxyfuel combustion power plant with CO_2_ capture presented by Stanger et al.^39^ analyzed the current technologies on the development of oxyfuel combustion for different kinds of coal-fired and gas turbine-based power plants. Retrofitting of a coal-fired power plant (CFPP) by a polymeric membrane and cryogenic distillation hybridization for different oxycombustion pathways were evaluated by Garcia et al.^43^ They reported that the polymeric membrane system is more efficient than cryogenic distillation regarding power consumption of the former in comparison to that of the latter.

Lim et al.^44^ developed a techno-economic analysis for 500 MWe ultrasupercritical steam power generation technology with circulating fluidized bed power plants with air combustion and oxycombustion in the presence of CO_2_ capture. Yu et al.^45^ investigated the plant performance of two flue gas recirculation modes, wet and dry modes, under different operating conditions for a 600 MW supercritical oxyfuel fluidized bed combustion power plant. Their results show that the dry-mode plant has a net power efficiency of 31.6%, while the wet-mode plant has a net power efficiency of 31.5%.

Aspen Plus software was used to model and simulate a cement plant for integrated oxyfuel combustion and power-to-gas technology.^46^ In former studies, the kinetic constants, absorption parameters, and energy requirement were commonly adjusted for simulations of MEA-based chemical absorption and fitting data with experiments^47−49^ Aspen Plus simulation for CO_2_ capture is widely used in research.^50,51^ Garcia et al.^52^ used commercial software for the validation of loading and temperature in a pilot plant. Miquel et al.^53^ simulated a model of the desorption column built in Aspen Plus using 30 wt % MEA. The mass transfer correlations and CO_2_ solubility were adjusted with experimental data on chemical absorption by Harbou et al.^54^ Joel et al.^55^ analyzed the intensified absorber for air combustion CO_2_ capture using a rotating packed bed with correlations in Aspen Plus rate-based model. They carried out the new correlations in visual Fortran as subroutines and linked them dynamically to the Aspen Plus rate-based model. Zhang et al.^56^ focused on the development of a dynamic model for the MEA-based CO_2_ capture process for an air combustion pulverized coal plant using the advanced linear model predictive control based on an Aspen Plus steady-state model. Nittaya et al.^57^ investigated a mechanistic dynamic model of an air combustion CO_2_ capture plant using the MEA absorption process. Bui et al.^58^ reviewed the dynamic modeling and optimization of flexible operation in air combustion CO_2_ capture plants. Cau et al.^59^ carried out the optimization of the process for energy penalization reduction with solvent regeneration through the retrofitting of a power plant with an MEA-based CO_2_ capture by full oxyfuel combustion that utilized cryogenic distillation air separation and their hybrid configuration.

Cau et al.^60^ assessed the commercial viability of partial oxyfuel combustion and examined simulation models of a carbon capture and storage (CCS)-equipped power plant. Their findings suggest that a greater CO_2_ content in flue gas with oxygen enrichment minimizes the energy penalty associated with solvent regeneration.

Cormos^61^ evaluated the key technical performances of oxyfuel combustion and postcombustion technologies. An in-depth analysis detected that the carbon capture energy penalties for coal-based supercritical oxycombustion plants are for postcombustion capture using alkanolamines. The cooling water needs of oxycombustion power plants are also reduced when compared with similar systems with postcombustion CO_2_ collection utilizing alkanolamines.

Yadav and Mondal^24^ provided a comprehensive review of the various novel oxyfuel technology configurations, compared in terms of energy penalty, auxiliary power consumption, CO_2_ product purity, and CO_2_ capture efficiency. All CCS technologies are associated with a substantial energy penalty. In comparison to other CCS approaches, oxyfuel combustion capture has a lower energy demand in the carbon capture section.^23^ According to the literature review, oxyfuel combustion is widely considered as a technically promising solution for carbon capture and storage to achieve net-zero emissions, compared to the conventional CCS approaches.

This work proposes a novel process configuration for carbon capture in oxyfuel combustion to deal with the problem related to high specific regeneration duty (SRD) associated with the CO_2_ absorption process by MEA solvent. It is compared with carbon capture in the case of air combustion technology, especially at high removal efficiency. In conventional oxyfuel technology, after the burning of fossil fuel in the presence of oxygen, a large proportion of flue gas is recycled into the combustion chamber to control the temperature of the combustion process. In the proposed configuration, the flue gas recycling takes place after the absorber of the carbon capture process. In such a case, the driving force of mass transfer in the absorption process is higher than that of the air combustion process. This phenomenon leads to smaller absorbent requirements, resulting in lower regeneration energy and MEA demands. To obtain a comprehensive technical comparison of air combustion and oxyfuel combustion methods, the carbon capture process with MEA is investigated for five different flue gases. In both methods, the same amount of CO_2_ capture is assumed at every capture rate. Then, the SRD and solvent consumption of the two technologies, that is, air and oxyfuel combustion, and the sensitivity analysis of the key operating parameters are compared.

## Model Development

### Parameters of Modeling and Configurations

**Figure 1:**
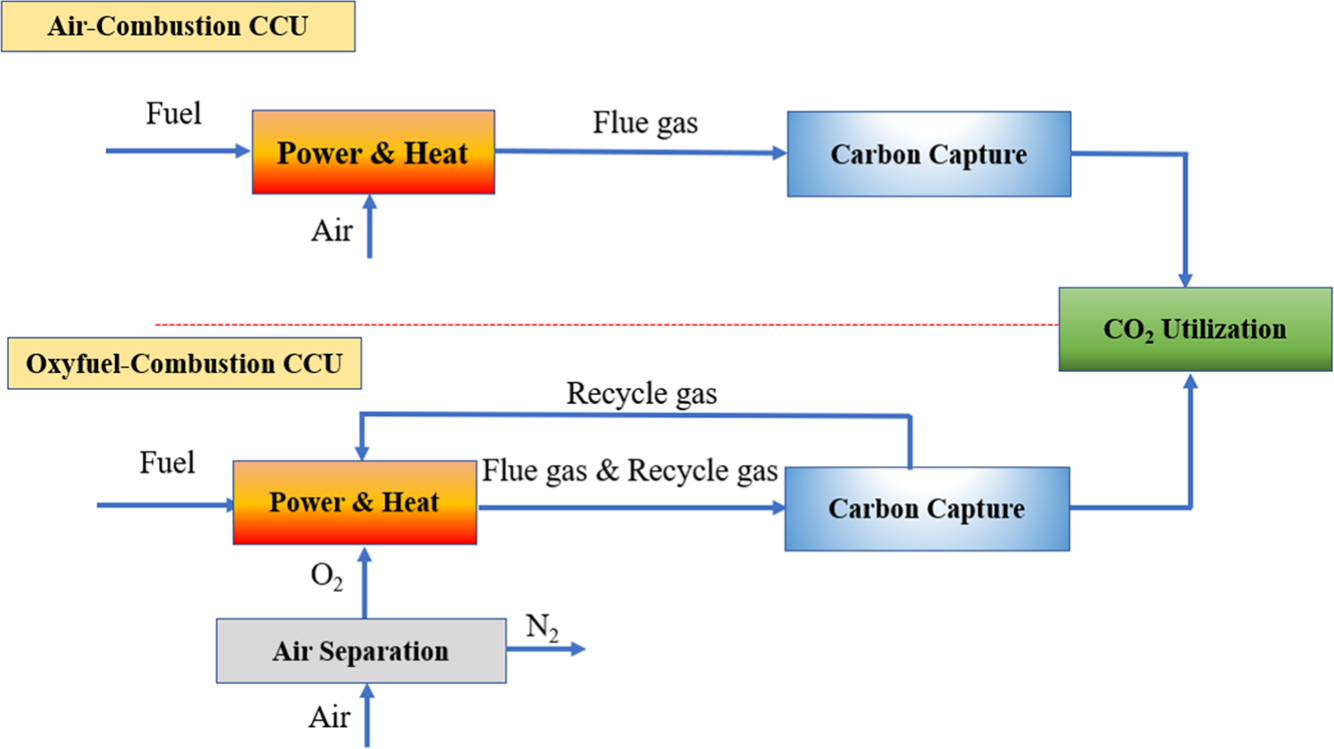
Schematic comparison of CO_2_ capture by the absorber–stripper process in air combustion and oxyfuel combustion.

Those oxyfuel technologies are studied where additional carbon dioxide is added as an inert gas to clean oxygen. Carbon capture processes are retrofitted to air combustion and oxyfuel combustion and modeled with Aspen Plus with chemisorption technology, considering 30 wt % aqueous MEA as an absorbent. According to the literature review, 30 wt % aqueous MEA is an industrially preferred solvent for CO_2_ removal from gas streams at around atmospheric pressure, and its relatively high loading capability affects the overall efficiency and effectiveness of the carbon capture process.^21,62,63^ The comparison of the schematic flowsheets of the two alternatives is presented in Figure 1. To compare carbon capture processes as part of air combustion and oxyfuel combustion technologies, only the amount of CO_2_ produced during the combustion process is captured. Although oxyfuel combustion modifies conventional chemical processes into more cost-effective, productive, and safer processes, it can also be beneficial as an alternative when there is no place for energy intensification in industrial sectors.^64^ It should also be mentioned that by using oxyfuel technologies, the formation of NO_*x*_ compounds can be avoided, providing additional environmental benefits by mitigating greenhouse gas emissions.^65,66^

Five different flue gases originating from different processes are selected, i.e., (i) gas-fired power plant;^67^ (ii) CFPP;^68,69^ (iii) coal-fired in a combined heat and power plant (Coal-CHP);^70,71^ (iv) aluminum production industry;^72^ and (v) cement manufacturing industry.^73,74^ The compositions and flow rates of the selected flue gases are given in Table 1. For the sake of comparison between air combustion and oxyfuel combustion, the compositions of flue gases are the same, but in the oxyfuel case, the nitrogen content is replaced with CO_2_ as an inert gas so that the special effects of pure oxygen can be avoided. The mass flow of the flue gases of oxyfuel combustion origin is higher because carbon dioxide’s molecular weight is higher than that of nitrogen. As a result, the density of the intake gas in oxyfuel combustion is substantially higher than in conventional air combustion. However, the amount of CO_2_ in the fossil fuel burning process is always the same.

**Table 1:** Flue Gas Compositions and Parameters

parameter	gas-fired	coal-fired	cement	aluminum	coal-CHP
temperature (°C)	40				
pressure (bar)	1.1				
air combustion					
flow rate (kg/s)	650				
composition	mol %				
H_2_O	8.00	8.21	7.20	1.00	12.11
N_2_	76.00	72.85	68.10	75.30	71.04
O_2_	12.00	5.36	2.30	20.70	3.36
CO_2_	4.00	13.58	22.40	3.00	13.49
oxyfuel combustion					
flow rate (kg/s)	921.39	895.76	871.40	913.16	900.00
composition	mol %				
H_2_O	8.00	8.21	7.20	1.00	12.11
N_2_	2.00	2.85	2.10	1.30	1.04
O_2_	12.00	5.36	2.30	20.70	3.36
CO_2_	78.00	83.58	88.40	77.00	83.49

**Figure 2:**
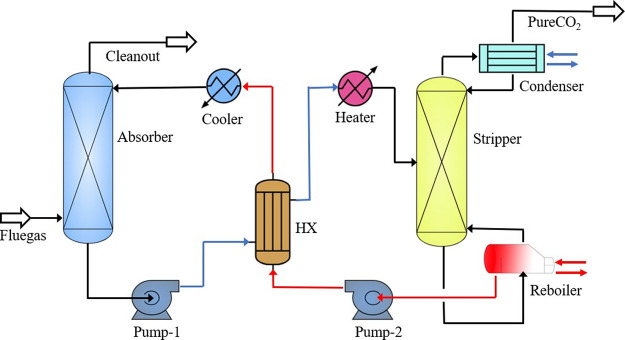
Configurations of carbon capture process based on chemical absorption technology.

The applied carbon capture process is presented in Figure 2. The accuracy of the model was verified using experimental data by Nagy and Mizsey.^21^ The flue gas is fed at the bottom of the absorber column and flows upward to have a countercurrent operation with the lean MEA fed at the top of the absorber. The rich MEA to be regenerated is fed into the stripper, which receives heat input from its reboiler. The rate-based model is applied to model the absorber–stripper system because it is more suitable than the equilibrium phase model.^75,76^ This model is based on two film theories compared to the equilibrium model, which is based on the theoretical number of equilibrium stages combined with Murphree’s efficiency concept. The two modeling methods have been checked with experimental data.^21^ Therefore, it can be concluded that the simulation of the absorber and desorber columns can be accurately and exactly modeled with the rate-based model since it gives a better prediction of the temperature and concentration profiles compared to the results of the equilibrium-stage model.^21,75^ As a result, for a detailed carbon capture process design, the rate-based model should be applied.^21^ In the rate-based model, CO_2_, N_2_, and O_2_ are selected as Henry components (solutes), where Henry’s law is applied. Henry’s constants are also specified for these components: water and MEA.

Table 2 shows the main input parameters of the columns and specifications that are used for model development in the absorber and stripper. Most of the specifications are recommended for the rate-based model of the CO_2_ capture process by Aspen Tech,^77^ and some of them are taken from the literature.^78,79^

**Table 2:** Aspen Plus Model Specifications of Absorber and Stripper Column for Air Combustion and Oxyfuel Combustion

specification	air combustion		oxyfuel combustion	
	absorber	stripper	absorber	stripper
number of stages	15	15	15	15
operating pressure	1.1 bar	2 bar	1.1 bar	2 bar
reboiler	none	kettle	none	kettle
condenser	none	partial vapor	none	partial vapor
packing type	Mellapak,Sulzer, Standard,250Y	Mellapak,Sulzer, Standard,250Y	Mellapak,Sulzer, Standard,250Y	Mellapak,Sulzer, Standard,250Y
parking height	20 m	18 m	22 m	18 m
packing diameter	15 m	12 m	18 m	12 m
interfacial area factor	1.2	1.5	1.2	1.5
heat transfer coefficient method	Chilton and Colburn	Chilton and Colburn	Chilton and Colburn	Chilton and Colburn
flow model	mixed	mixed	mixed	mixed

### Chemistry of the MEA–H_2_O–CO_2_ Reaction System

For modeling the MEA–H_2_O–CO_2_ system, different thermodynamic assumptions are applied in the liquid and the vapor phases. The presence of dissolved CO_2_ in the form of carbonate and carbamate in the liquid phase has an electrolyte behavior; consequently, the electrolyte nonrandom two liquid thermodynamic model is used for the liquid phase.^80^ As the ions are not assumed to be present in the vapor phase, the SRK EoS model is selected for the description of the thermodynamic behavior of this phase.^81^

The important chemical reactions taking place in a system are described in this section (eqs 1–5).^82^

MEA protonation:
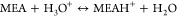
1

Carbonate formation:
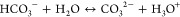
2

Hydrogen carbonate formation:
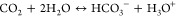
3

Carbamate formation:
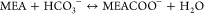
4

Water dissociation:
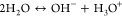
5

The chemical equilibrium constant *K*_eq_ is calculated according to eq 6. In this equation, *T* is the absolute temperature, and A–D are the constants listed in Table 3.
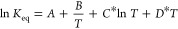
6

**Table 3:** Reaction Equilibrium Constants Used in This Work

reaction no.	*A*	*B*	*C*	*D*	ref
1	216.05043	–12437.7	–35.4819	0	(55)
2	231.46543	–12092.1	–36.7816	0	(55)
3	–3.038325	7008.357	0	–0.003134	(83)
4	–0.52135	–2545.53	0	0	(83)
5	132.8988	13445.9	–22.4773	0	(83)

The equilibrium reactions are applied to all of the modules in the process simulation except for the absorber and stripper columns. These two columns are controlled at each stage kinetically. The equilibrium reactions 3 and 4 are replaced with rate model eqs 7 and 8 and reverse reactions 9 and 10.
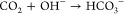
7
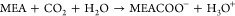
8
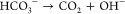
9
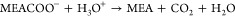
10

The rate of kinetic reactions is described by the power-law expression represented in eq 11, where *r* is the rate of reaction, *k* is the pre-exponential factor, *T* is the absolute temperature, *R* is the gas constant, and *E* is the activation energy.^84^ These values are summarized in Table 4.
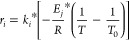
11

**Table 4:** Rate Constant Values in Absorptive CO_2_ Capture^85^

parameter	reaction 7	reaction 8	reaction 9	reaction 10
*k*_*j*_	4.3200 × 10^13^	9.7700 × 10^10^	2.3800 × 10^17^	2.7963 × 10^20^
*E*_*j*_ (kJ/mol)	5.85	41.20	113.10	72.02
*T*_0_ (K)	298	298	298	298

### Key Performance Indicators for Process Analysis

The following key performance indicators are specified in this research to conduct performance evaluation and comparative assessment for air and oxyfuel combustion technologies:

### Removal Efficiency of the Process

The removal efficiency is defined as the percentage of CO_2_ captured by the process. This indicator is calculated according to eq 12.
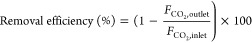
12Where *F*_CO2,inlet_ and *F*_CO2,outlet_ represent the inlet and outlet CO_2_ flow rates in the absorber unit, respectively. Six different CO_2_ removal efficiencies in the range of 80–97% are considered for air and oxyfuel combustion cases. Regarding oxyfuel combustion, 100% removal efficiency is also considered. The removal efficiency indicates only the amount of CO_2_ that comes from the fossil fuel combustion process. In the case of oxyfuel combustion, the excess CO_2_ is handled as a recycled inert gas. Therefore, the amount of carbon capture is identical for each removal efficiency in the two alternatives.

### Carbon Dioxide Loading

In conventional carbon capture configurations, carbon dioxide loading indicates the performance of the capture process, which reflects the efficiency of the mass transfer taking place in the absorber. According to eq 13, the CO_2_ loading is defined as the ratio of the total mole quantity of apparent CO_2_ to the total mole quantity of apparent MEA in the solvent. There are two CO_2_ loading definitions in the absorption process: (i) the lean loading, determined for the stream leaving the bottom of the desorber and (ii) the rich loading, calculated for the solvent after the absorption process.
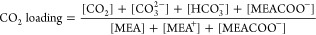
13

### SRD

The regeneration of the absorbent is an energy-intensive operation that influences the whole carbon capture process. Therefore, SRD is considered a dominant indicator to evaluate the total performance of the process. SRD is defined in eq 14.
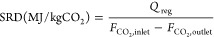
14where *Q*_reg_ notes the regeneration duty in the reboiler and *F*_CO2,inlet_ and *F*_CO2,outlet_ is defined as the inlet and outlet CO_2_ flow rates in the absorber. Only that amount of CO_2_ is considered to come from the fossil combustion process.

## Results and Discussion

### Solvent Consumption of Oxyfuel and Air Combustion

Table 5 presents the absorbent flow rates and the liquid/gas ratio values at different carbon capture rates for the five flue gases selected (Table 1). In the oxyfuel combustion cases, two carbon capture rates are shown: (i) the capture rate of the CO_2_ of the combustion origin and (ii) the real capture rate that includes the CO_2_ recycled as an inert compound. In the cases of both alternatives, the solvent flow rate increases if a higher carbon capture rate is required.

**Table 5:** Solvent Flow Rates in Different Flue Gases

air combustion										
flue gas	gas-fired		coal-fired		cement		aluminum		coal-CHP	
removal efficiency %	solvent flow (kg/s)	L/G ratio	solvent flow (kg/s)	L/G ratio	solvent flow (kg/s)	L/G ratio	solvent flow (kg/s)	L/G ratio	solvent flow (kg/s)	L/G ratio
80	610.51	0.94	1,435.68	2.20	2,215.11	3.40	491.17	0.75	1,457.87	2.24
85	616.74	0.94	1,456.75	2.24	2,248.99	3.45	495.57	0.76	1,479.08	2.27
90	623.00	0.95	1,477.70	2.27	2,282.89	3.51	500.08	0.76	1,500.08	2.30
92	625.44	0.96	1,486.26	2.28	2,296.73	3.53	501.89	0.77	1,508.57	2.32
95	629.33	0.96	1,499.69	2.30	2,318.02	3.56	504.71	0.77	1,521.95	2.34
97	631.55	0.97	1,508.13	2.32	2,332.68	3.58	506.66	0.77	1,530.87	2.35

oxyfuel combustion															
flue gas	gas-fired			coal-fired			cement			aluminum			coal-CHP		
CO_2_ removal efficiency %	CO_2_ real removal efficiency %	L/G ratio	solvent flow (kg/s)	CO_2_ real removal efficiency %	L/G ratio	solvent flow (kg/s)	CO_2_ real removal efficiency %	L/G ratio	solvent flow (kg/s)	CO_2_ real removal efficiency %	L/G ratio	solvent flow (kg/s)	CO_2_ real removal efficiency %	L/G ratio	solvent flow (kg/s)
80	4.10	0.52	481.81	13.01	1.38	1,240.29	20.27	2.34	2,043.12	3.11	0.40	369.16	12.92	1.44	1,298.31
85	4.35	0.52	487.31	13.81	1.40	1,259.54	21.54	2.38	2,081.54	3.31	0.40	373.49	13.73	1.46	1,317.86
90	4.61	0.53	493.18	14.62	1.42	1,279.39	22.81	2.42	2,114.59	3.50	0.41	377.59	14.54	1.48	1,336.79
92	4.71	0.53	495.74	14.92	1.43	1,286.72	23.31	2.44	2,126.81	3.58	0.41	379.11	14.86	1.49	1,344.73
95	4.87	0.54	499.16	15.43	1.44	1,298.02	24.07	2.46	2,146.35	3.70	0.41	381.44	15.34	1.50	1,356.95
97	4.97	0.54	501.36	15.76	1.45	1,306.26	24.58	2.47	2,159.18	3.78	0.41	383.27	15.67	1.51	1,364.58
100	5.12	0.54	505.03	16.25	1.47	1,317.87	25.34	2.49	2,178.12	3.89	0.42	385.71	16.15	1.52	1,376.50

Since in the case of oxyfuel combustion the nitrogen content of the air is replaced with carbon dioxide, the total carbon dioxide flow rate and its concentration in the absorber tower in this technology are significantly higher than those of air combustion processes. The higher CO_2_ content results in a lower absorbent requirement, and the CO_2_ removal efficiencies are lower than those of the air combustion cases. The reduction of solvent is calculated to be approximately 24, 20, 13, 10, and 7% for the aluminum industry, gas-fired, coal-fired, coal-CHP, and cement industry, respectively.

In the case of oxyfuel combustion, 100% carbon capture can also be completed, where the real carbon capture rates range between 4 and 26%. The flue gases of the oxyfuel combustion have a much higher CO_2_ concentration over the whole capture process,^36^ which means a higher mass transfer gradient. Therefore, the absorbent and energy requirement values are much lower than those in the air combustion case, demonstrating a more beneficial capture process.

### Comparison of Energy Consumptions

**Figure 3:**
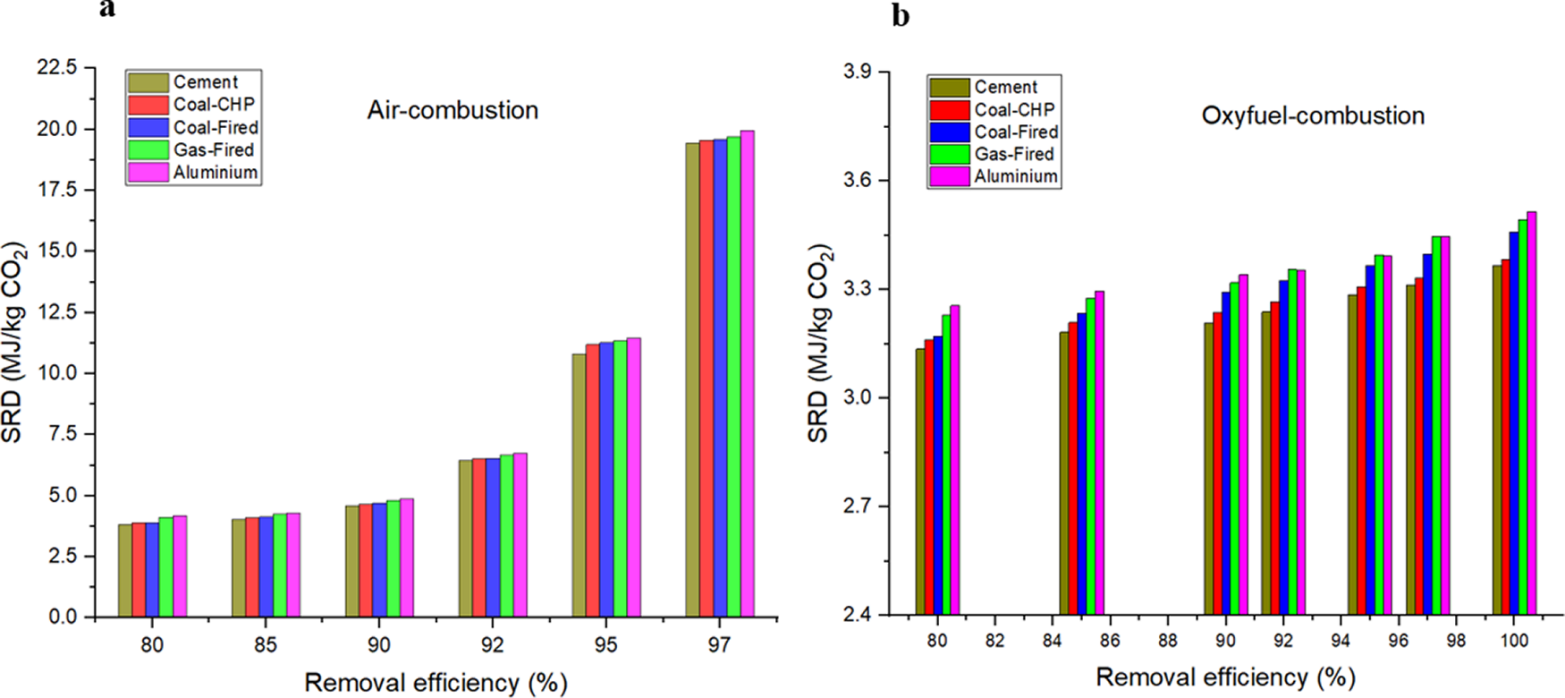
SRD variation with removal efficiency in (a) air combustion and (b) oxyfuel combustion.

SRD variation with removal efficiency is demonstrated in Figure 3. The change in the energy consumption values is demonstrated by the values of the specific regeneration duties (SRD) for the two alternatives. It is obtained that

- The oxyfuel combustion case needs less energy input and a smaller SRD due to the higher driving force for the mass transfer.
- If the capture rate is increasing, the SRD is also increasing.
- The increase in SRD is exponential in the cases of air combustion.

**Figure 4:**
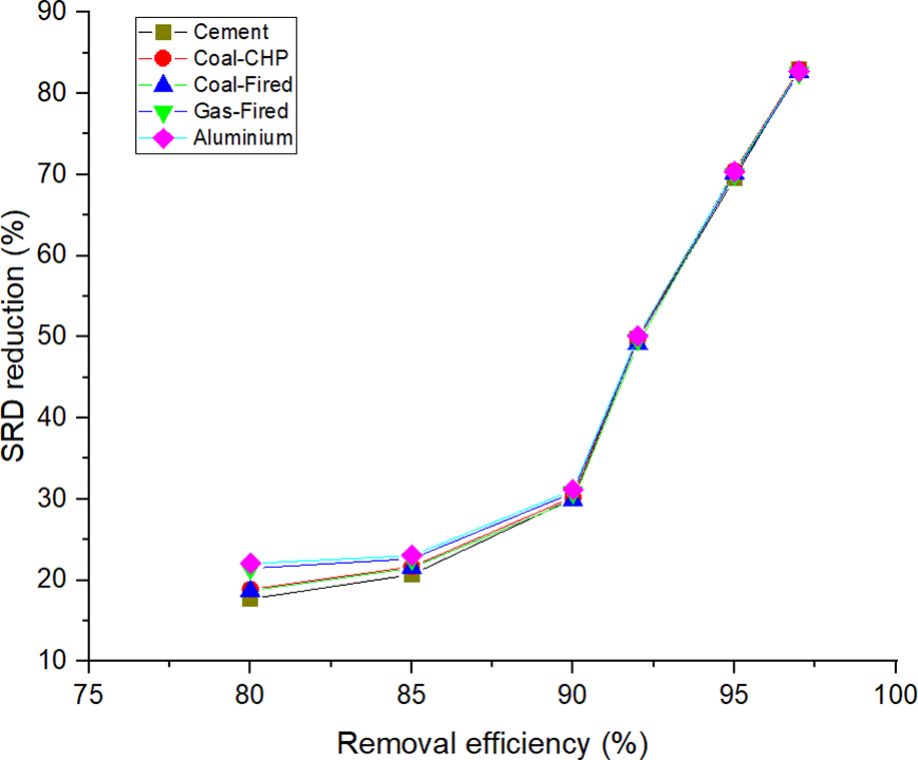
SRD reduction with the application of oxyfuel combustion.

The result of SRD reduction (calculated in eq 15) in the two processes for all of the simulated flue gases is illustrated in Figure 4. SRD_AC_ and SRD_OXY_ indicate the SRD for air combustion and oxyfuel combustion, respectively.
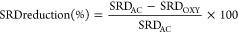
15

The results show that higher CO_2_ removal efficiency results in higher energy concumption but also that in the case of oxyfuel combustion, the energy requirement is significantly smaller. The reason for the smaller energy consumption is due to the higher driving force of the mass transfer in the oxyfuel combustion than that of the air combustion technologies. The CO_2_ mass transfer between gas and liquid in the absorber tower is investigated according to the two-film theory. The mass transfer rate across the gas boundary is calculated by eq 16. *K*_G_ and *A* are the binary mass transfer coefficient and mass transfer surface area, respectively, which are fixed for both technologies. So, only the concentration difference can influence the mass transfer. A higher carbon dioxide concentration in the flue gas (*y*) results in a higher concentration difference and a higher mass transfer rate.
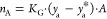
16

According to the novel configuration proposed in this research, flue gas recycling takes place after the carbon capture process (Figure 1) in the absorption tower. In this case, a higher CO_2_ concentration can be carried out in the absorber, and only that amount of CO_2_ is captured that originates from the burning process. The excess amount of flue gas is recycled.

To show the differences in the CO_2_ concentration (*y*) in the absorber of the captured process, the ratios of the CO_2_ concentrations in the cases of the oxyfuel combustion and the air combustion technologies are calculated and compared (eq 17, Table 6).
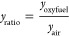
17

**Table 6:** *y*_ratio_ of the CO_2_ Concentration in the Inlet and Outlet Gaseous Streams in the Absorber Tower

removal efficiency (%)	gas-fired		coal-fired		cement		coal-CHP		aluminum	
	inlet *y*_ratio_	outlet *y*_ratio_	inlet *y*_ratio_	outlet *y*_ratio_	inlet *y*_ratio_	outlet *y*_ratio_	inlet y_ratio_	outlet *y*_ratio_	inlet *y*_ratio_	outlet *y*_ratio_
80	19.5	93.8	6.1	26.9	3.9	15.2	6.2	26.9	25.6	125.7
85	19.5	124.6	6.1	36.3	3.9	19.9	6.2	35.	25.6	170.0
90	19.5	186.2	6.1	52.3	3.9	29.6	6.2	54.4	25.6	254.2
92	19.5	248.0	6.1	67.7	3.9	36.2	6.2	70.4	25.6	323.6
95	19.5	371.0	6.1	111.8	3.9	60.7	6.2	116.3	25.6	507.8
97	19.5	741.0	6.1	167.0	3.9	100.1	6.2	173.7	25.6	887.5

Table 6 shows the *y*_ratio_ values for different CO_2_ removal efficiencies and every flue gas investigated. The inlet ratios are identical per definition; however, the huge numbers of outlet ratios show the beneficial feature of this kind of oxyfuel technology. After the *y*_ratio_ values are seen, it can be explained why the carbon capture process is more attractive in the case of proper oxyfuel technology. Less absorbent can be applied, and the energy requirement is also lower.

### Effect of Removal Efficiency on CO_2_ Lean Loading

**Figure 5:**
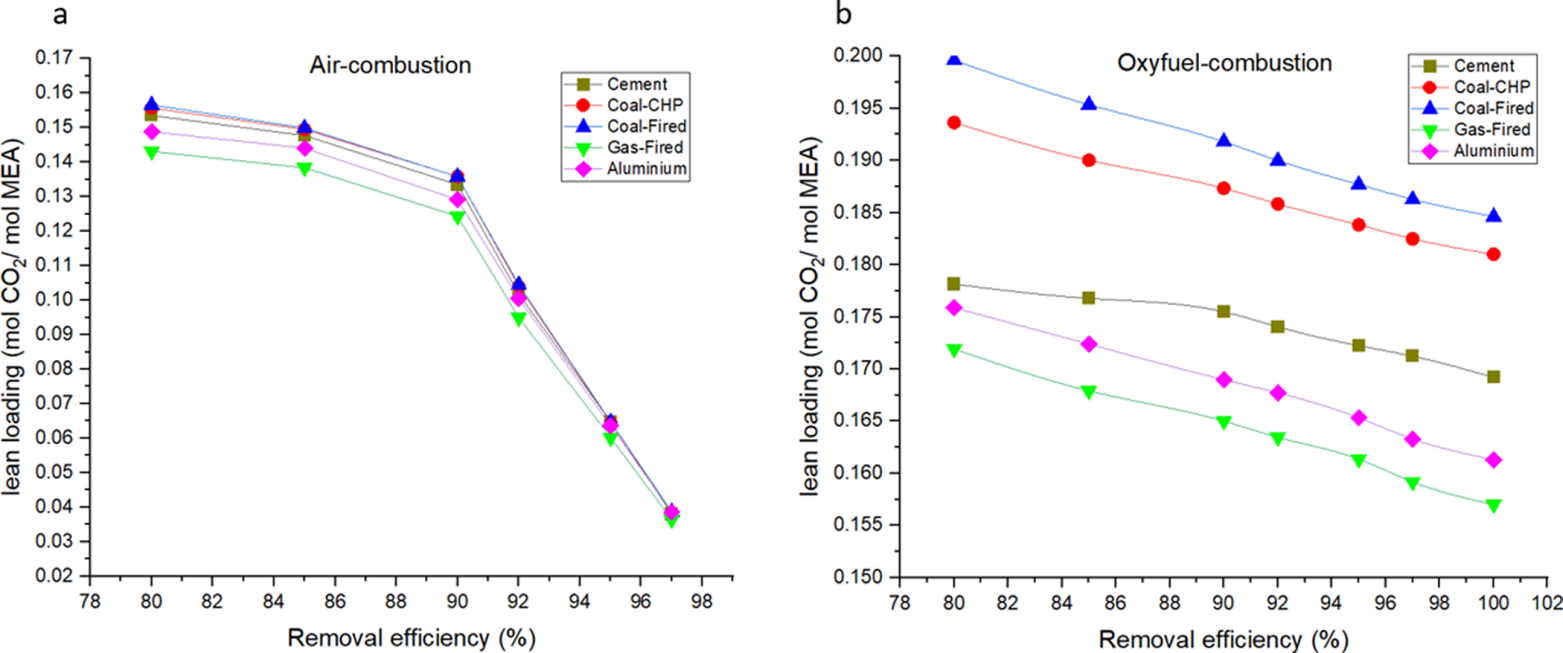
Variation of lean loading with removal efficiency in (a) air combustion as well as (b) oxyfuel combustion.

Figure 5 shows the corresponding results related to the lean loading variation due to the removal efficiency change in air combustion as well as oxyfuel combustion for the five simulated flue gases. CO_2_ lean loading decreases as removal efficiency increases. However, in the case of air combustion, lean loading decreases more quickly for removal efficiencies of >90% (Figure 5a). The reason for this is that guaranteeing the necessary driving force for the CO_2_ mass transfer requires a low lean loading. Consequently, the content of CO_2_ in the lean solvent stream rapidly decreases. In the oxyfuel combustion case, the lean loading also decreases with higher removal efficiency; however, there is no rapid reduction in this trend, as the driving force of this technology is relatively high compared to air combustion (Figure 5b).

### Impact of Lean Loading on Specific Reboiler Duty

The lean loading shows the ratio of CO_2_ and MEA in the absorbent, leaving the desorber unit. Low lean loading is needed if a high CO_2_ removal efficiency is required and/or the CO_2_ concentration in the flue gas is low. These two effects are connected. The low lean loading requires higher reboiling, which translates into higher specific reboiler duty.

**Figure 6:**
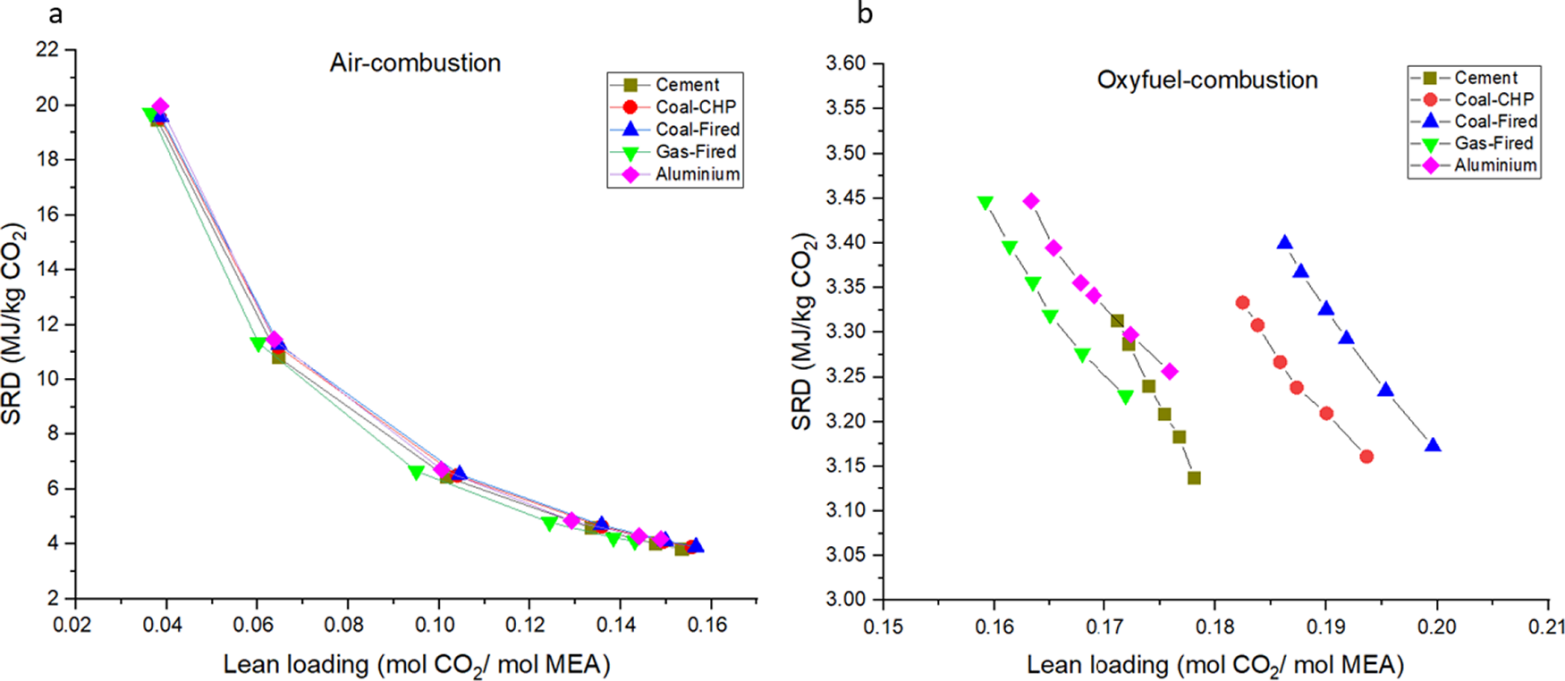
Effect of lean loading variation on the specific regeneration duty in (a) air combustion as well as (b) oxyfuel combustion.

The effects of lean loading variation on the SRD for the two investigated technologies are listed in Figure 6. The reduction of lean loading needs energy, that is, an SRD increment. This change in air combustion is more extensive than that of oxyfuel combustion due to the significantly different CO_2_ concentrations in the flue gases. It can be seen that the oxyfuel combustion is advantageous over air combustion. It can also be seen that the lowest lean loading is required in the case of gas-fired combustion and the highest is required in the coal-fired case.

### Impact of Oxyfuel Combustion Substitution with Air Combustion on Liquid/Gas Ratios

**Figure 7:**
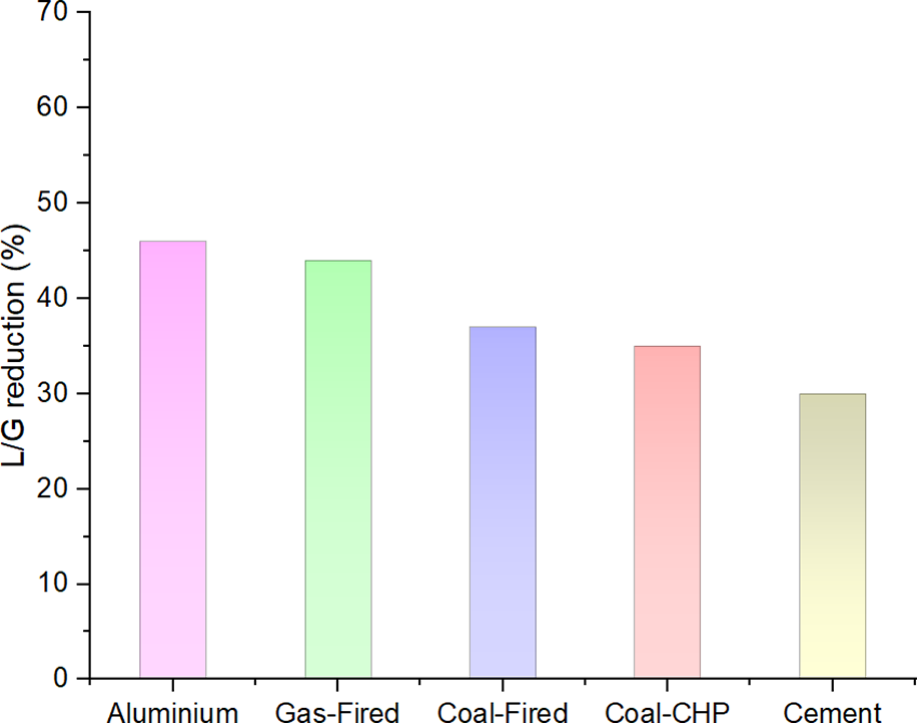
L/G reduction for the simulated flue gases by substitution of air combustion with oxyfuel combustion.

Figure 7 shows the L/G reduction if air combustion is replaced with oxyfuel combustion. In eq 18, the absorbent gas ratios, that is, (L/G)_AC_ and (L/G)_OXY_ indicate the L/G ratios in air and oxyfuel combustion, respectively. As a general trend, in each technology, by increasing the solvent flow for the sake of a higher carbon capture rate, the L/G ratios are to be increased. The highest L/G reduction is reported for the flue gas with the lowest CO_2_ content (aluminum), and the flue gas from the cement industry has the lowest L/G reduction due to its highest CO_2_ content among the case studies. This agrees with the conclusion obtained before that the lower CO_2_ composition needs more absorbent and lower lean loading so that the driving force for mass transfer can be guaranteed.

The reduction of L/G by using oxyfuel combustion instead of air combustion shows the potential of this technology to capture the same amount of CO_2_ with a smaller amount of solvent, which is inserted into the process as a liquid stream.
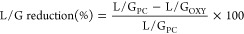
18

## Conclusions

As mobility will shift to electric vehicles, which require electric power, and electricity is still largely produced on a fossil basis, carbon capture must be seriously considered and possibly promoted. According to this chemical engineering study, oxyfuel technologies can show more chemical attraction for CO_2_ capture than air-based ones. The application of clean oxygen with artificially backmixed carbon dioxide as an inert to replace nitrogen is studied, which is usually applied in retrofit cases. Such oxyfuel technology has a higher carbon dioxide composition in the flue gases, resulting in a higher driving force for mass transfer in the absorber. As demonstrated by five different flue gases from oxyfuel technologies and compared to the air combustion alternative, the oxyfuel technology is much better from the carbon capture point of view. Carbon capture requires less energy, especially if more than 90% carbon dioxide removal is designed than that of the air combustion case. Even the total amount, 100%, of carbon dioxide from the combustion process can be easily captured. Besides the lower energy consumption, the carbon capture process can work at a lower absorbent/flue gas ratio and with a smaller amount of absorbent than the air-based processes. The energy savings can be as high as 84%, and, on the other hand, no NO_*x*_ emissions are produced in the oxyfuel combustion processes, further reducing greenhouse gas emissions. The application of this oxyfuel process, replacing the inert effect of nitrogen with carbon dioxide, can improve the advantages of carbon capture and facilitate the fulfillment of environmental prescriptions. In this study, only a technical feasibility study of oxyfuel combustion was completed since the economic features are highly country-dependent. In the future, further research is required to evaluate economic, cost, or long-term reliability before practical implementation in the application area.
